# Whole genome sequence of the *Treponema pallidum* subsp. *pallidum* strain Amoy: An Asian isolate highly similar to SS14

**DOI:** 10.1371/journal.pone.0182768

**Published:** 2017-08-07

**Authors:** Man-Li Tong, Qiang Zhao, Li-Li Liu, Xiao-Zhen Zhu, Kun Gao, Hui-Lin Zhang, Li-Rong Lin, Jian-Jun Niu, Zhi-Liang Ji, Tian-Ci Yang

**Affiliations:** 1 Zhongshan Hospital, Medical College of Xiamen University, Xiamen, China; 2 Institute of Infectious Disease, Medical College of Xiamen University, Xiamen, China; 3 State Key Laboratory of Stress Cell Biology, School of Life Sciences, Xiamen University, Xiamen, Fujian, P.R. China; Oregon State University, UNITED STATES

## Abstract

*Treponema pallidum* ssp. *pallidum* (*T*. *pallidum*), the causative agent of the sexually transmitted disease syphilis, is an uncultivatable human pathogen. The geographical differences in *T*. *pallidum* genomes leading to differences in pathogenicity are not yet understood. Presently, twelve *T*. *pallidum* genomes are available to the public, all of which are American in origin and often co-infect patients with human immunodeficiency virus (HIV). In this study, we examined the *T*. *pallidum* subsp. *pallidum* strain Amoy, a syphilis pathogen found in Xiamen, China. We sequenced its genome using Illumina next-generation sequencing technology and obtained a nearly (98.83%) complete genome of approximately 1.12 Mbps. The new genome shows good synteny with its five *T*. *pallidum* sibling strains (Nichols, SS14, Mexico A, DAL-1, and Chicago), among which SS14 is the strain closest to the Amoy strain. Compared with strain SS14, the Amoy strain possesses four uncharacterized strain-specific genes and is likely missing six genes, including a gene encoding the TPR domain protein, which may partially account for the comparatively low virulence and toxicity of the Amoy strain in animal infection. Notably, we did not detect the 23S rRNA A2058G/A2059G mutation in the Amoy strain, which likely explains the sensitivity of Amoy strain to macrolides. The results of this study will lead to a better understanding of the pathogenesis of syphilis and the geographical distribution of *T*. *pallidum* genotypes.

## Introduction

The spirochete *Treponema pallidum* ssp. *pallidum* (*T*. *pallidum*) is the cause of the sexually transmitted disease syphilis. However, the exact pathogenesis of this disease is poorly understood.[1] The difficulty of continuously cultivating *T*. *pallidum in vitro* prevents the use of common genetic approaches to study these organisms.[2] In 1998, the first complete genome sequence of *T*. *pallidum* (Nichols strain) was completed, providing a valuable source for identifying treponemal virulence factors, targets for molecular typing, and candidates for potential vaccine development.[3] Subsequently, independent whole-genome sequencing projects produced the genome sequences of twelve *T*. *pallidum* strains from different sources.[1, 4–6] These studies revealed that the genomic differences between individual strains were minor.[7] However, these complete *T*. *pallidum* genome sequences are all from America, including the Nichols (U.S. Navy, 1912),[3] Chicago (U.S. Chicago, 1951),[8] Mexico A (U.S. Mexico, 1953),[5] SS14 (U.S. Atlanta, 1976),[1] and DAL-1 (African American woman, 1991) strains. [6] Little genomic information for *T*. *pallidum* strains from areas outside of North America, such as Asia or China, has been reported thus far.

Previous studies have proposed that *T*. *pallidum* strains from different areas around the world possess varied genotypes, leading to differences in pathogenicity.[9–11] Molecular typing of *T*. *pallidum* conducted in the United States, South Africa, Portugal, Scotland, Canada, Madagascar, Ireland, Colombia, and China showed that 27 of the most common subtypes exhibited substantial geographic variation and genetic diversity.[11] Sequencing of ribosomal RNA (rRNA) operons indicated that different rRNA spacer patterns (Ile/Ala and Ala/Ile) appeared to be randomly distributed in the treponemal strains, regardless of species/subspecies classification, sampling time, and geographical source.[12] Therefore, as syphilis is a worldwide epidemic disease, it would be impossible to understand the genetics underlying the ability of *T*. *pallidum* to evade the host immune system without obtaining genomic information on *T*. *pallidum* strains from different prevalence areas. Previously sequenced *T*. *pallidum* strains from the Americas have shown little variation in their gene sequences.[7] Therefore, whole genome sequencing of *T*. *pallidum* strains from China will help to identify differences in variants due to geographic disparities.

In this study, we conducted whole genome sequencing on the *T*. *pallidum* subsp. *pallidum* Amoy strain. This strain was first isolated from a patient with primary syphilis in Xiamen, China in 2011. To obtain a sufficient sample for sequencing, we infected rabbits with the Amoy strain. However, to avoid possible genetic changes in the harvested syphilis, we did not continuously pass the Amoy strain in rabbits. The infection of rabbits lasted for an average of 18 days before treponeme harvest, which differs from the infection periods for other strains.[13, 14] Our preliminary animal experiments revealed that the Amoy strain has some unique characteristics. For example, the Amoy strain shows relatively low toxicity in animal infections Compared with other strains, which hints at a potential difference in genetics. Therefore, genome sequencing of the Amoy strain will help to determine the genetic variations between the Amoy strain and other *T*. *pallidum* isolates and provide insight to reveal the genetic differences that underlie their different mechanisms of pathogenesis.

## Materials and methods

### Ethics statement

The Institutional Ethics Committee of Zhongshan Hospital, Medical College of Xiamen University, reviewed and approved this study. We performed the study in compliance with national legislation and the Declaration of Helsinki guidelines, and we obtained written patient consent according to institutional guidelines before performing the experiments. The study protocol employed seronegative New Zealand white male rabbits (3 to 4 months old) for *T*. *pallidum* propagation. All rabbit experiments strictly followed the parameters outlined by the Institutional Animal Care and Use Committee (IACUC) and were approved by the animal experimental ethics committee of the Medical College of Xiamen University.

### *T*. *pallidum* strain propagation and DNA isolation

We isolated the *T*. *pallidum* Amoy strain from the chancre of a primary syphilis patient in Xiamen Zhongshan hospital on June 23, 2011. The strain was then inoculated intratesticularly into rabbits as previously reported.[3, 15] Briefly, two rabbits were sedated with acepromazine via intramuscular injection at 1 to 3 mg/kg body weight according to the IACUC protocol and were then injected with 5 × 10^7^ *T*. *pallidum* cells per testis. After 7 days of inoculation, the rabbits were checked daily for disease progression. Approximately 18 days after infection, the animals were euthanized (intravenous injection with pentobarbital at 90 mg/kg body weight) according to IACUC guidelines and secured on a rabbit restraining board at peak orchitis to harvest the greatest number of *T*. *pallidum* specimens before the onset of immune clearance. The testes were aseptically removed and minced in 10 ml of saline with 10% normal rabbit serum per testis for approximately 10 min. Then, the suspensions were washed and centrifuged at least two times (7 min at 500 x g) to remove host cellular debris, after which the supernatant was centrifuged at 12,000 x g for 30 min to pellet *T*. *pallidum*. Sequentially, we re-suspended the spirochetes in 1 ml of phosphate-buffered saline (PBS) and purified the spirochetes using discontinuous Hypaque-M 75% gradients (Renografin-60, Hunan Hansen Pharmaceutical Co., LTD, China) as previously described.[3, 16] DNA extraction was performed using the QIAGEN Genomic-tip kit (Qiagen Inc., Chatsworth, CA) according to the manufacturer’s instructions. To remove contamination by rabbit DNA, we treated the purified *T*. *pallidum* liquid with 0.02 mg/ml DNase I (Sigma Chemical Co. St. Louis, MO, USA) before DNA extraction. The extracted DNA was stored at -20°C. [14]

### Whole-genome sequencing and assembly

Library construction and sequencing were performed by the Beijing Genomics Institute (BGI) on a Genome Analyzer IIx System (Illumina Inc., San Diego, CA, USA) in 90-base pair (bp) paired-end mode. Before proceeding with genome assembly, we performed a quality control (QC) evaluation on the raw sequencing data using NGS-QC to exclude low-quality reads, if they failed to satisfy the criterion of a PHRED quality score of 20 for 70% of the read length.[17] This step was followed by additional taxonomic analysis using the Kraken[18] program to remove potential contaminated reads, using all microbial genomes in GenBank as a reference. We adopted the *de novo* assembly software IDBA_UD [19] to assemble the clean reads into contigs, using k-mers from 30 to 60. Subsequently, we used SSPACE3.0 [20] to scaffold the pre-assembled contigs, embedding Burrows-Wheeler Aligne for sequence alignment, with minimum error of 0.25 and an insert size of 481. GapFiller was then used to close gaps within and between scaffolds using the same parameters as SSPACE. [21] The synteny of the *T*. *pallidum* Amoy strain against its sibling strain *Treponema pallidum subsp*. *pallidum* SS14 was determined using Mauve. Via synteny analysis, we estimated the missing sequences (gaps) in the Amoy strain at the same time (S1 Table).[22] Subsequently, we employed the ABACAS program to order and orient the scaffolds into a complete genome and filled the gaps between scaffolds with Ns.[23]

### Genome annotation, comparisons, and functional annotation

We annotated the genome using the NCBI PGAAP pipeline, [24] tagging the genes with an A4W95 prefix for the Amoy strain. We performed genomic comparisons of the Amoy strain against five other published genomes of *T*. *pallidum* strains,[25] including Nichols (NC_021490.2), SS14 (NC_021508.1), Mexico A (NC_018722.1), DAL-1 (NC_016844.1), and Chicago (NC_017268.1). We built phylogenetic relationships based on these six different *T*. *pallidum* strains, adopting *Treponema pallidum subsp*. *pertenue str*. Gauthier as an outlier, using the online tool REALPHY 1.10. The REALPHY program uses the maximum likelihood method PhyML [26] to infer the tree with default parameters of a read length of 50 and a seed length of 22. [27] Pan-genome analysis was conducted with GET_HOMOLOGUES software (v2.0.20), using COGtriangles[27] and OrthoMCL[28] algorithms, with parameters of sequence coverage ≥ 75%, an E-value ≤ 1e-05 and sequence identity ≥ 1%. We carried out functional annotations using KEGG BlastKOALA, which compares encoded amino acid sequences against the Kyoto Encyclopedia of Genes and Genomes (KEGG) database. We also used InterProScan5 to assign GO terms to each CDS.[29] Eventually, we deposit the genome sequences in the GenBank database under accession number CP015162 and annotation ID NC_ CP015162.1.

## Results and discussion

### The whole genome of the *T*. *pallidum* Amoy strain

**Fig 1:**
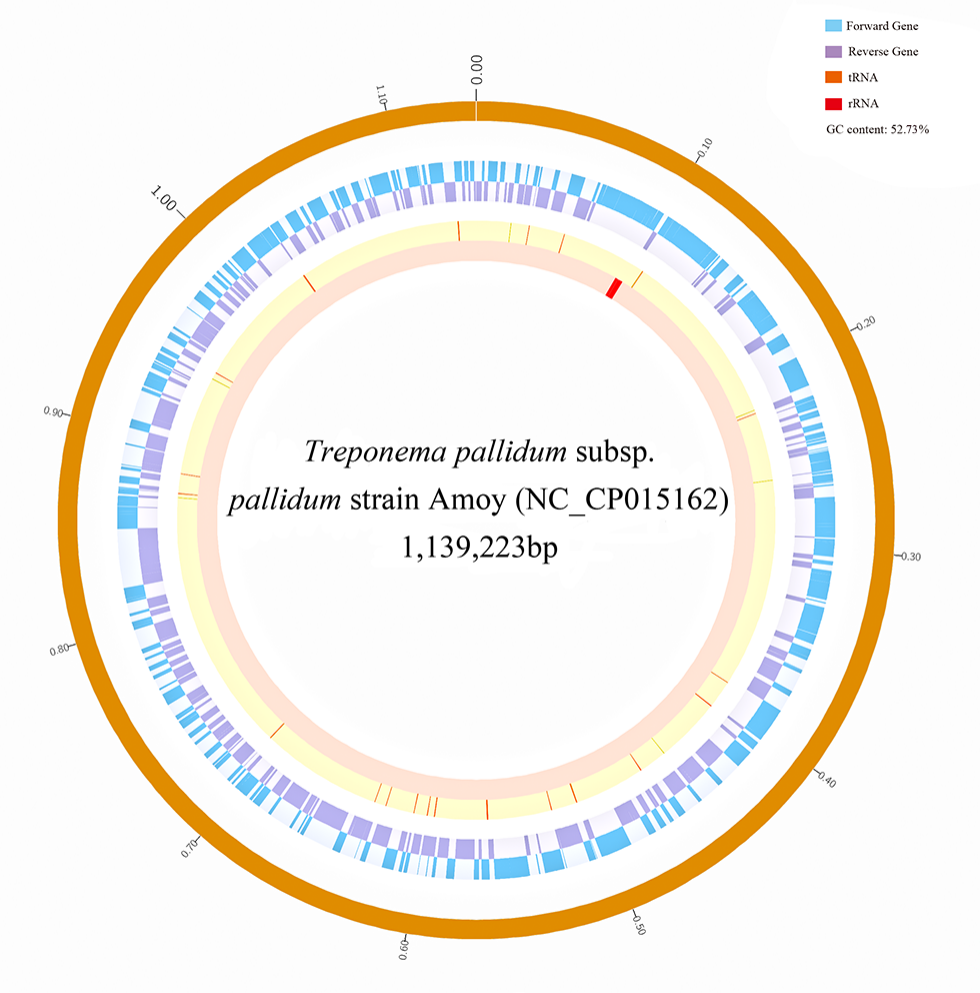
The circular genome of *T*. *pallidum* Amoy.

Genome sequencing of the Amoy strain on the Illumina HiSeq 2000 platform yielded about 2.82G base pair raw paired-end reads. Approximately 367 Mb of clean reads passed the QC and contamination checks. Compared with the genome size (1.14 Mb) of the sibling strain *T*. *pallidum* SS14, the average sequencing depth was approximately 300X, which is sufficient for high-quality genome assembly. After employing a computational pipeline for assembly, re-scaffolding, gap closing, and scaffold ordering, we eventually obtained a draft circular genome of 1,139,223 base pairs, which consisted of 15 scaffolds (98.83%) and approximately 1.17% Ns (Fig 1).

Table 1 shows the basic genomic statistics of the Amoy strain (Table 1). Excluding Ns, the average G + C content of the Amoy strain genome is 52.73%, which is in agreement with other *T*. *pallidum* strains.[5] In total, the genome encodes 1,063 genes, including 995 coding CDSs, 43 tRNAs, 3 rRNAs, 3 ncRNA, and 19 pseudogenes. Of these genes, 724 encode functional proteins. The functional categorization of these CDS by COG (Clusters of Orthologous Groups) analysis revealed that most of the sequences are involved in translation, ribosomal structure, biogenesis, cell wall synthesis, replication, and other metabolic processes (S1 Fig). Additional GO enrichment analysis showed that the genes of the Amoy strain mainly participate in catalytic activity, metabolism, nucleobase, nucleoside, nucleotide, nucleic acid metabolism, transferase activity, and other metabolic processes (S2 Fig).

**Table 1:** Genome statistics of the *T*. *pallidum* Amoy strain.

Attribute	Value
Total bases (bp)	1,139,223
As	24.25%
Ts	23.01%
Gs	25.14%
Cs	27.59%
(A + T)s	47.26%
(G + C)s (without Ns in gaps)	52.73%
Ns	0.01%
Genes (total)	1,063
CDS (total)	1,014
Genes (coding)	995
CDS (coding)	995
Genes (RNA)	49
rRNAs	3 (5S, 16S, 23S)
tRNAs	43
ncRNAs	3
Pseudogenes	19

### Genomic comparisons of the Amoy strain with its *T*. *pallidum* siblings

**Fig 2:**
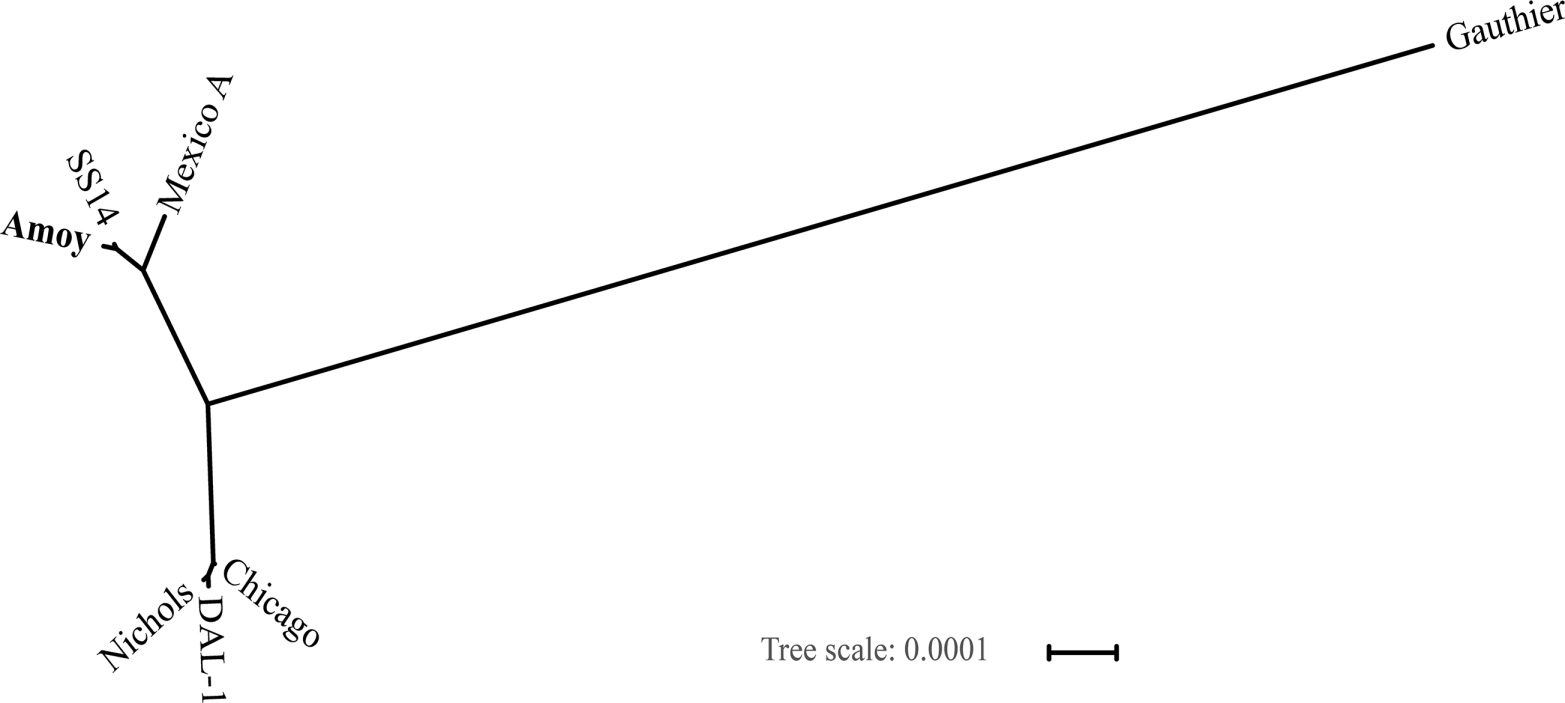
Genome-based classification of *T*. *pallidum* strains. A phylogenetic tree was built based on the Amoy, DAL-1, Chicago, Mexico A, Nichols, and SS14 genomes, adopting Gauthier (CP002376.1) as an outlier, using REALPHY with default parameters for the maximum likelihood method.

To determine the phylogenetic position of the Amoy strain, we compared the new genome to five known *T*. *pallidum* genomes (Chicago, DAL-1, Mexico A, Nichols, SS14) according to their sequence similarities, using a close strain, *Treponema pallidum subsp*. *pertenue str*. Gauthier, as an outlier. Genome clustering assigned the six *T*. *pallidum* genomes to two groups (Fig 2). The Nichols-like group consists of Chicago, DAL-1 and Nichols, and the SS14-like group consists of Amoy, Mexico A and SS14. This result is consistent with previous phylogenetic studies on *T*. *pallidum* strains.[30, 31] Indeed, most *T*. *pallidum* strains that cause infections throughout the world are SS14-like strains, including our Amoy strain.[7] Therefore, we carried out co-synteny analysis of the Amoy strain genome by referring to the SS14 strain, which revealed that thirteen genes, including *arp*, *tpr*C, *tpr*D, *tpr*E, *tpr*G, *tpr*I, *tpr*J, 5s rRNA, 16s RNA, 23s RNA, tRNA-Ala, tRNA-Ile and a hypothetical protein, were likely located in gap regions of the Amoy strain genome.

**Fig 3:**
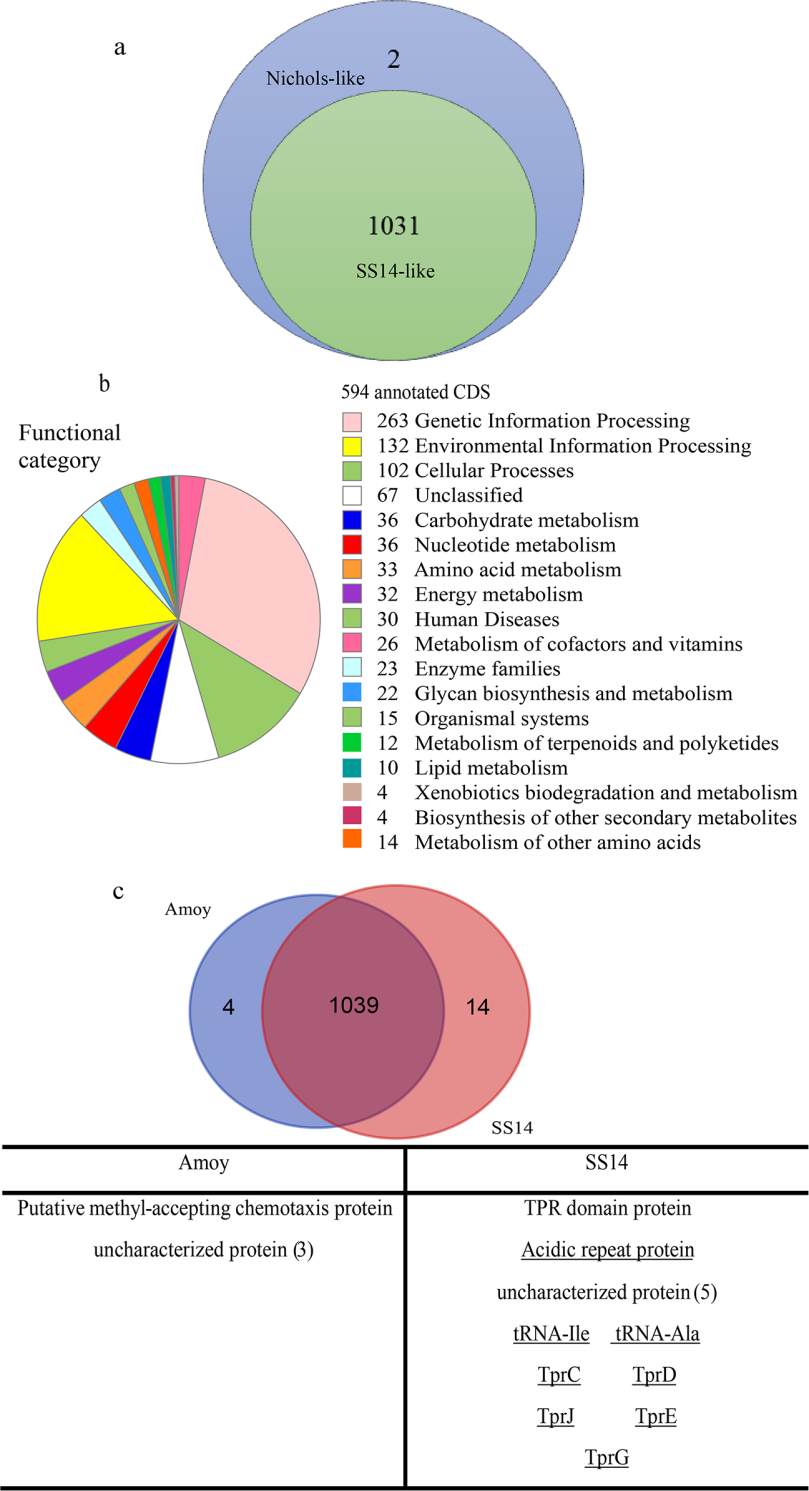
**a**: **Comparison of the genes of Nichols-like strains (Chicago, DAL-1, Nichols) and SS14-like strains (Amoy, Mexico A, SS14)**. A total of 1,031 genes were shared by all strains, except for two uncharacterized genes that were Nichols-like strain specific. **b**: **Functional annotation of the genome using BlastKOALA**, 594 of 975 genes in Amoy with known functions assigned by KEGG ontology. **c: Comparison of genes between the Amoy strain and the SS14 strain.** According to co-synteny analysis, the underlined genes are likely located at gap regions in Amoy.

Through pan-genome analysis, we found that 1,031 genes were common to all of the *T*. *pallidum* strains, except for two genes specific to the Nichols-like group (Fig 3A). Both of these genes encode uncharacterized proteins (WP_014342799.1 and WP_014342776.1); however, they may serve as indicators for accurate classification of *T*. *pallidum* strains. Compared with the other five strains, two genes were found to be Amoy specific, and four genes have likely been lost in the Amoy strain, excluding seven genes in the gap region of the Amoy strain genome (Table 2). In addition, we performed a close comparison between the Amoy strain and the SS14 strain (Fig 3C). In addition to the 1,039 mutual genes, four genes were Amoy specific (including the two Amoy-specific genes in Table 2), and six genes were SS14 specific, excluding the potential genes in the gap. The four Amoy-specific genes included a chemotaxis protein and three uncharacterized proteins. The six SS14-specific genes consisted of a gene encoding a TPR domain protein and five uncharacterized proteins. The TPR domain protein is a tpr-like gene which are candidate virulence factor that has received intense research scrutiny in treponemal infections over the last decade. The loss of the TPR domain protein could partially explain why Amoy shows comparatively low virulence and toxicity in animal infections; it also provides insight for differentiating *T*. *pallidum* strains in future genotyping studies.

**Table 2:** Comparison of the genes of the Amoy strain against other five *T*. *pallidum* strains.

Amoy	SS14, Mexico A, Nichols, DAL-1, Chicago
uncharacterized proteins (2)	uncharacterized proteins (4)
	tRNA-Ile tRNA-Ala
	tprD tprC
	tprG tprE
	tprJ

The underlines genes are likely located at gap regions in the Amoy strain genome according to co-synteny analysis.

### Macrolide resistance of the Amoy strain

For years, more than 94% of the clinical isolates identified based on the enhanced CDC typing system (tpr/arp/tp0548) belonged to the SS14-like group.[7] The reason for this discrepancy is not yet known; a possible explanation is the macrolide resistance of SS14-like strains.[7] In the middle of the last century, Nichols-like strains were frequently identified in the syphilis-carrying population when antibiotics were first developed for the treatment of infection.[7] Subsequently, *T*. *pallidum* strains mutated in response to selective pressure from widespread antibiotic use, and different antibiotic-resistant strains, such as SS14, appeared in the population. Fortunately, syphilis has not yet developed resistance to penicillin.[32] However, two mutations (A2058G or A2059G in 23S rRNA) conferring resistance to macrolides have been identified, possibly resulting from azithromycin treatment of sexually transmitted diseases.[33] A previous study indicated that in Hunan, China, up to 97.5% of samples harbor the A2058G mutation,[34] and in Shanghai, China, up to 97.5% of syphilis isolates harbor the A2058G mutation.[35] In the present study, considering that 23S rRNA is likely located at the gap region of the Amoy strain genome, we used PCR and Sanger sequencing methods to re-sequence the Amoy strain 23S rRNA sequence, which revealed neither the A2058G nor the A2059G mutation. As there are no data indicating the prevalence or geographic distribution of macrolide-resistant strains of *T*. *pallidum* in the Xiamen area, it is not clear whether the A2058G/A2509G wild-type of the Amoy strain is an accidental or a prevalent strain in Xiamen. Population genetic analysis of sufficient syphilis patients in the Xiamen area is therefore desired in the future.

## Conclusion

In this study, we sequenced the genome of the *T*. *pallidum* Amoy strain, providing the first genome sequence of a clinical syphilis isolate from China. According to analysis of genomic similarity, the Amoy strain is mostly closely related to the SS14-like group. At the same time, we also illustrated the genomic differences of the *T*. *pallidum* Amoy strain compared with other strains from various host populations and different geographic regions, identifying two uncharacterized proteins specific to the Amoy strain. Unlike current prevalent isolates, we did not detect a 23S rRNA A2058G/A2059G mutation in the Amoy strain, which partially explains the absence of macrolide resistance in the Amoy strain. Overall, the sequenced genome of the *T*. *pallidum* Amoy strain will lead to a better understanding of different types of pathogenesis of *T*. *pallidum* strains and will contribute to the goal of achieving syphilis eradication.

## Supporting information

S1 Fig Functional category of CDS in the *T*. *pallidum* Amoy strain. (TIF)

S2 Fig Gene ontology enrichment analysis of genes in the Amoy strain. (TIF)

S1 Table Gap information for the Amoy strain in comparison with the reference strain SS14. (XLSX)
