# Supplementary figures and images for: Whole genome sequence of the Treponema pallidum subsp. pallidum strain Amoy: An Asian isolate highly similar to SS14
Source: PLoS One. 2017 Aug 7;12(8):e0182768. doi: 10.1371/journal.pone.0182768 (PMC5546693; doi:10.1371/journal.pone.0182768)

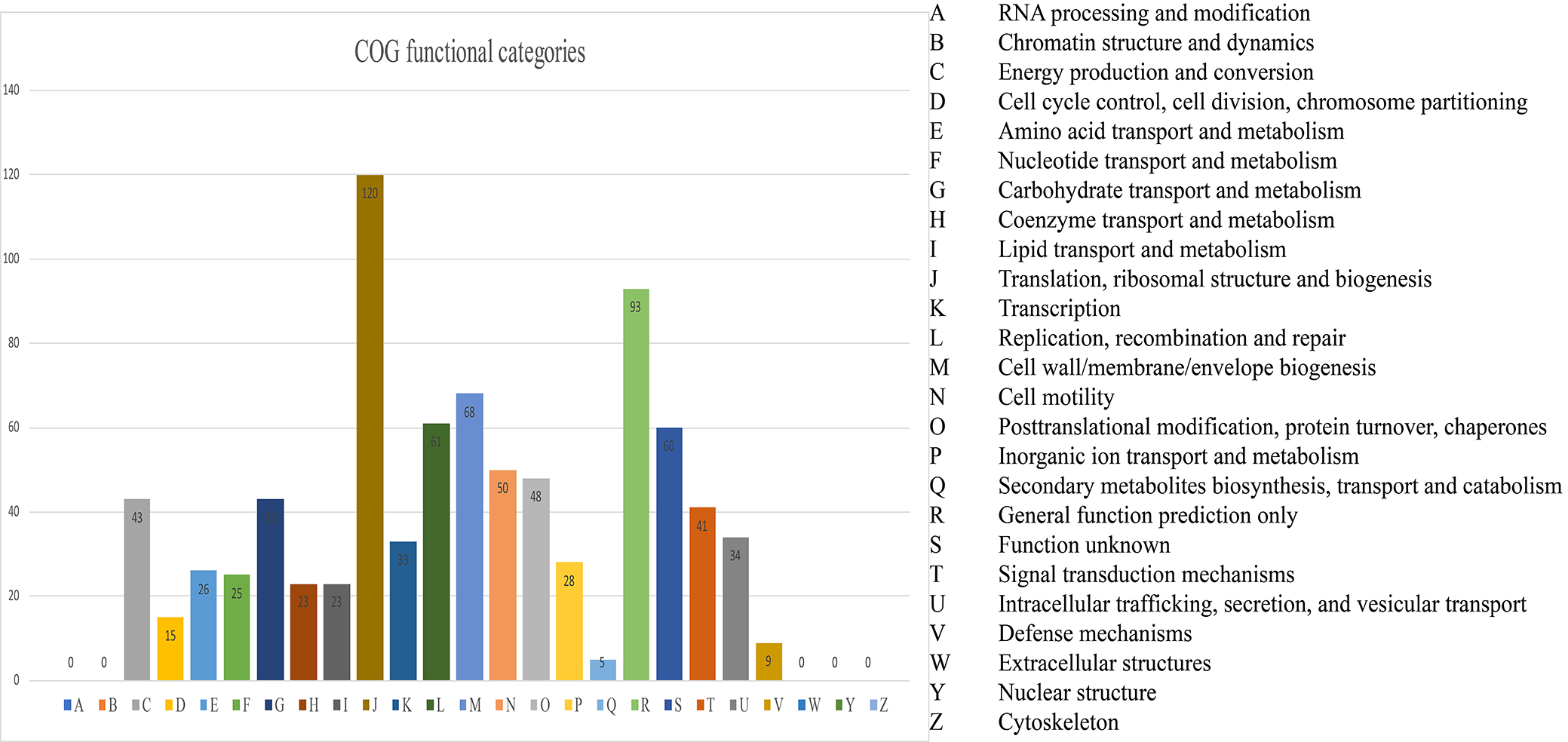

Supplement: S1 Fig — (TIF) [file pone.0182768.s001.tif]

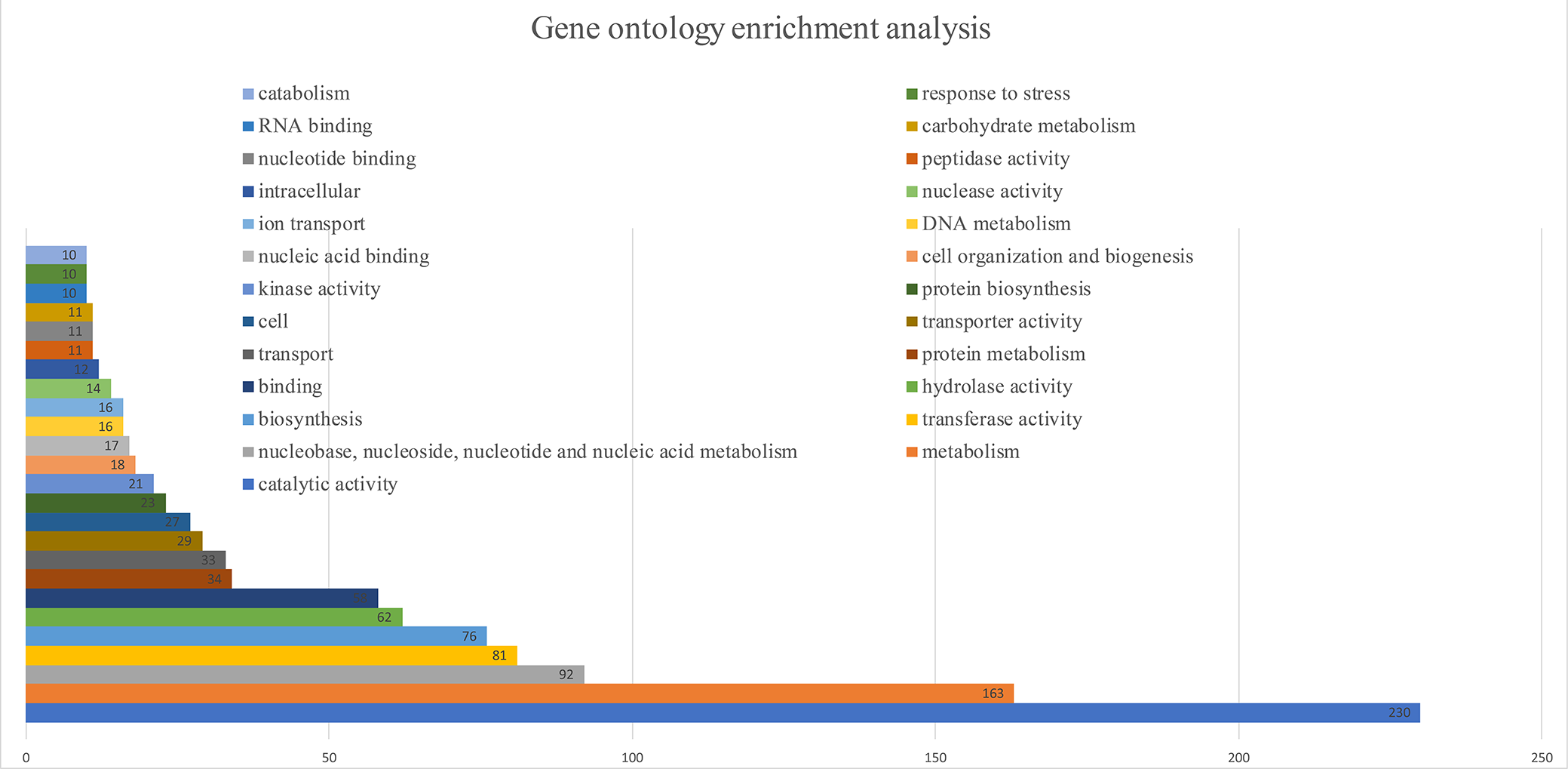

Supplement: S2 Fig — (TIF) [file pone.0182768.s002.tif]
